# Effect of Chemical Cross-Linking on Compatibility and Laboratory Performance of SBS/PE/EVA Ternary Composite Modified Asphalt

**DOI:** 10.3390/ma19071476

**Published:** 2026-04-07

**Authors:** Hong Zhang, Cheng Wang, Yiming Chen, Ning Li, Tao Zhou, Yu Mao, Yan Zhang

**Affiliations:** 1 Key Laboratory of Environment-Friendly Composite Materials of the State Ethnic Affairs Commission, College of Chemical Engineering, Northwest Minzu University, Lanzhou 730124, China; 13704987089@163.com (C.W.); cyiming_material@163.com (Y.C.); 19960558595@163.com (N.L.); 2 Gansu Provincial Biomass Function Composites Engineering Research Center, College of Chemical Engineering, Northwest Minzu University, Lanzhou 730124, China; 3 Key Laboratory for Utility of Environment-Friendly Composite Materials and Biomass in University of Gansu Province, College of Chemical Engineering, Northwest Minzu University, Lanzhou 730124, China; 4 Gansu Province Research Center for Basic Sciences of Surface and Interface Chemistry, College of Chemical Engineering, Northwest Minzu University, Lanzhou 730124, China; 5 Gansu Provincial General Station of Agricultural Ecology and Resource Conservation Technology Extension, Lanzhou 730000, China; 18241898531@163.com; 6 Gansu Provincial Transportation Research Institute Group Co., Ltd., Lanzhou 730030, China; myjkjt@126.com (Y.M.); 13188326507@163.com (Y.Z.)

**Keywords:** styrene-butadiene-styrene (SBS), orthogonal test, rheological properties, composite modified asphalt, microcosmic analysis

## Abstract

In response to the shortcomings still observed in polyethylene (PE)/ethylene-vinyl acetate (EVA)/styrene-butadiene-styrene (SBS) composite modified bitumen regarding storage stratification and low-temperature performance, this paper further introduces furfural extract, elemental sulphur, stabilisers and Z-6036 into this ternary system, and employs orthogonal design to screen the additive ratios. Tests were conducted on conventional physical properties, rotational viscosity, dynamic shear rheology and bending beam rheology, focusing on the material’s temperature sensitivity, rheological behaviour, low-temperature creep resistance and phase characteristics. The modification effects were analysed using fluorescence microscopy, scanning electron microscopy and infrared spectroscopy. Compared with the control group composed of 4% PE, 4% EVA and 2% SBS, the samples obtained from the orthogonal design showed an increase in elongation at 5 °C ranging from 52.5% to 213.9%; the difference in softening points decreased from 35.2 °C to a minimum of 0.1 °C, indicating improved storage stability. The temperature sensitivity of all sample groups was reduced, with the optimal group achieving a VTS of −0.4413, representing a 46.7% improvement over the control group. At −12 °C, the m-values of all nine orthogonal samples were higher than those of the control group, with seven groups reaching m ≥ 0.3, indicating improved low-temperature stress relaxation capability. A comprehensive analysis of the experimental results indicates that the selected chemical additives are beneficial for optimising the dispersion state and compatibility of the SBS/PE/EVA ternary modified bitumen, whilst also balancing rheological properties and low-temperature crack resistance to a certain extent. Microscopic and spectroscopic analyses further suggest that internal interactions within the system have been enhanced and the phase distribution has become more uniform; however, the current evidence is insufficient to conclusively determine that a specific form of chemical cross-linking reaction has occurred.

## 1. Introduction

Asphalt is the most commonly used binder in flexible pavements; however, under conditions of heavy traffic, significant temperature fluctuations and long-term service, conventional asphalt often struggles to simultaneously meet multiple requirements, such as resistance to deformation at high temperatures, resistance to cracking at low temperatures and durability. To improve its performance in service, polymer modification and rubber modification have become key technical approaches in the research and engineering applications of asphalt materials [1,2,3,4]. In recent years, the utilisation of waste plastics, waste rubber and their composites in the field of modified bitumen has attracted sustained attention [5,6,7,8,9,10,11,12]. This is not only linked to the need for resource recovery and environmental sustainability, but is also closely related to the application potential demonstrated by these materials in terms of high-temperature stability, fatigue performance and long-term durability.

Among the numerous modification systems, thermoplastic components—typically represented by PE—are characterised by their wide availability, relatively controllable costs, and significant stiffening effects; however, when used alone, they are often associated with issues such as insufficient compatibility, an increased risk of stratification during storage, and limited low-temperature toughness [2,3,4,5,6,7,8]. Consequently, blending thermoplastic components with elastomers or components possessing superior dispersion capabilities has become a key approach for balancing multiple road performance characteristics. Existing studies generally indicate that the modification of multi-component polymers is not a simple summation of the effects of individual components; rather, their final performance is closely related to phase distribution, interfacial interactions, preparation processes and formulation design [9,10,11,12]. In other words, whether a composite system can achieve synergistic performance depends crucially on the stability of the internal structure and the compatibility between the components.

Empirical studies have validated this approach across various composite systems. Research on EVA/rubber compound-modified asphalt reported improved conventional, rheological and microstructural behaviour [9]. Waste tyre rubber (WTR) combined with reclaimed low-density polyethylene was also found to reduce penetration and phase angle while increasing softening point, rotational viscosity and complex modulus [10]. Recycled polyethylene and crumb rubber (CR) were reported to improve the anti-ageing properties and thermal stability of asphalt binders [11]. In addition, LDPE/SBS composite modification has been shown to enhance rheological performance and formulation stability in polymer-modified binders [12]. Systematic evaluation of waste plastic/rubber modified asphalt further indicated that polymer ratio, pretreatment method and microstructural mechanisms significantly affect storage stability and overall service performance. Overall, multi-component polymer modification offers a viable approach to balancing diverse service requirements; however, the extent of performance synergy between different systems remains subject to the combined constraints of formulation and structural evolution processes.

Based on the above findings, it remains necessary to conduct more targeted formulation screening and mechanistic analysis regarding the PE/EVA/SBS ternary composite modification system, with a view to improving storage stability and compatibility whilst also enhancing low-temperature performance. At the same time, the mode of action of chemical additives in such systems, their optimal dosage, and their correlation with macroscopic road performance characteristics remain to be further clarified. To this end, this study introduced furfural extract, elemental sulphur, stabilisers and Z-6036 into the PE/EVA/SBS ternary composite modified bitumen system, and employed an orthogonal experimental design to optimise the formulation ratios; Building on this, a systematic evaluation of the macroscopic performance and microstructural characteristics was conducted using conventional performance tests, rotational viscosity tests, dynamic shear rheology tests, flexural beam rheology tests, as well as fluorescence microscopy, scanning electron microscopy and Fourier Transform Infrared Spectroscopy analysis [13,14]. Because elemental sulphur and related additives require careful handling, their use in this study was restricted to controlled laboratory conditions with adequate ventilation, personal protective equipment and proper waste management.

## 2. Experimental Methods

### 2.1. Raw Materials

This study selected 90# road petroleum bitumen, commonly used in the Gansu region, as the base bitumen and designated it as BA-90,the properties of which are shown in Table 1. Low-density polyethylene (PE) was supplied by Ningbo Qingteng Plastic Co., Ltd. (Ningbo, Zhejiang, China), and ethylene-vinyl acetate copolymer (EVA) was supplied by Ningbo Shuyou Plasticization Co., Ltd. (Ningbo, Zhejiang, China). Styrene-butadiene-styrene block copolymer (SBS), grade T6302H with a styrene content of 30%, was produced by Dushanzi Petrochemical Company (Karamay, Xinjiang, China). The vinyl acetate content of EVA was 40%. Furfural extract oil (FEO) was supplied by Shandong Longshengda New Material Technology Co., Ltd. (Zibo, Shandong, China). Elemental sulphur powder and silane coupling agent Z-6036 were both purchased from Aladdin (Shanghai, China). The stabiliser, supplied by Gansu Luqiao Shanjian Technology Co., Ltd. (Lanzhou, Gansu, China), was selected according to the SBS modified asphalt stabiliser reported in Ref. [15]. The main performance parameters of PE and EVA are presented in Table 2, and all raw materials were stored in a dry and sealed condition prior to sample preparation.

### 2.2. Preparation of Modified Asphalt Samples

**Figure 1:**
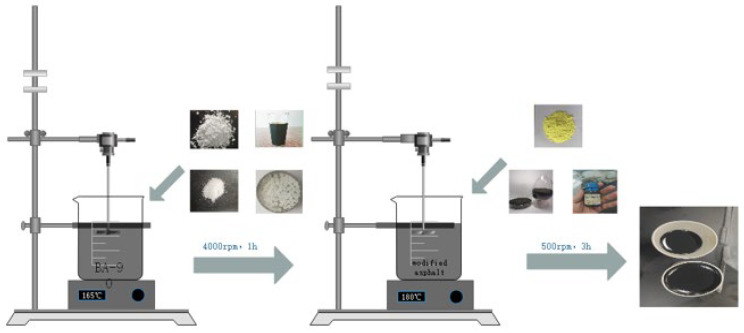
Flow diagram of SBS/PE/EVA ternary composite modified asphalt sample preparation.

The preparation of SBS/PE/EVA ternary composite modified bitumen was carried out in the sequence of preheating of the matrix, melting and dispersion of polymers, and post-addition homogenization of additives. First, 500 g of matrix bitumen was weighed and heated to 140 ± 5 °C until fully fluidized, after which 2% SBS, 4% PE and 4% EVA were slowly added. The system was then heated to 160 °C and pre-sheared at 2000 r/min for 5 min to achieve preliminary dispersion of the three polymers within the bitumen phase. After pre-mixing, FEO was added, and the mixture was continuously sheared at 180 °C and 4000 r/min for 60 min to further refine the polymer phase. Finally, the stabiliser, elemental sulfur and Z-6036 were added, and the mixture was stirred at 180 °C and 500 r/min for 3 h. This final step was not intended as continued high-shear mixing; rather, the extended low-speed mixing time was used to ensure homogeneous dispersion of the subsequently added additives throughout the pre-homogenised SBS/PE/EVA binder and to obtain a macroscopically uniform material before testing. Although such prolonged thermal exposure may introduce some short-term ageing, all binders were prepared under the same protocol; therefore, the rheological results in this study are interpreted mainly in terms of relative differences among formulations. The specific preparation process is shown in Figure 1.

### 2.3. Orthogonal Design of Experiments

Taking into account the synergistic effects of FEO, elemental sulphur, stabilisers and Z-6036 in a ternary modification system, this study employed an L9(3^4^) orthogonal design to compare the effects of different factors and their levels on asphalt performance within a limited number of experiments, and to screen for the optimal formulation. The four factors are denoted as A, B, C and D, where A represents FEO, B represents elemental sulphur, C represents the stabiliser, and D represents Z-6036. The levels of each factor are shown in Table 3, and the test combinations are listed in Table 4. The ranges of each additive were determined by preliminary tests: FEO from 2% to 4%, elemental sulphur from 0.08% to 0.12%, stabiliser from 0.15% to 0.25%, and Z-6036 from 0.02% to 0.10%. To distinguish between the ‘polymer blending effect’ and the ‘effect of subsequent additives’, two control samples were additionally prepared: 4P+4E and 4P+4E+2S. The former contains only 4 wt% PE and 4 wt% EVA, whilst the latter adds 2 wt% SBS to this base. Neither control sample contains FEO, elemental sulphur, stabiliser, or Z-6036.

### 2.4. Conventional Properties Test

In order to compare the basic viscosity, temperature response and low-temperature extensibility of different samples at a macro level, this paper tested three conventional parameters: penetration, softening point and elongation at 5 °C. The penetration test was conducted at 25 °C, the softening point was determined using the ring-and-ball method, and the elongation test was carried out at 5 °C. The respective tests were conducted in accordance with ASTM D5/D5M-20, ASTM D36/D36M-14 and ASTM D113-17 [16,17,18].

### 2.5. Rotational Viscosimeter Test

To characterise the flow behaviour of the samples under high-temperature conditions and their sensitivity to temperature changes, a rotational viscometer was used to measure the viscosity of each group of bitumen at 135, 155 and 175 °C, with three replicate tests conducted at each temperature [19]. After obtaining the temperature–viscosity data for the three groups, the viscosity–temperature sensitivity coefficient VTS was calculated using Equation (1) to compare the effectiveness of different formulations in regulating high-temperature flow behaviour. In the equation, η represents the bitumen viscosity, T represents the test temperature, A represents the intercept of the linear fit, and VTS represents the slope of the fit.

(1) $$\log\left[\log\left(\eta \right)\right]\left\{\begin{array}{cc} <1.0945, & \log\left[\log\left(\eta \right)\right]=A+\mathrm{VTS}\times \log(T)\\ \geq 1.0945, & \log\left[\log\left(\eta \right)\right]=1.0945 \end{array}\right.$$

### 2.6. Dynamic Shear Rheometer (DSR) Test

The dynamic shear rheology (DSR) tests were conducted on bituminous binders prepared in accordance with Section 2.2 and not subjected to RTFOT or PAV ageing treatments, with a focus on investigating the influence of formulation differences on the initial viscoelastic response. Temperature-sweep tests were conducted in accordance with ASTM D7175 [20], with a test temperature range of 46–76 °C, temperature intervals of 6 °C, and a loading frequency of 1.59 Hz (10 rad/s). During the tests, the complex modulus G* and phase angle δ were recorded, and the rutting factor G*/sinδ was calculated from these values to evaluate the material’s resistance to deformation at high temperatures [21].

Building on the temperature scanning tests, multi-stress creep recovery (MSCR) tests were further employed to compare the permanent deformation behaviour and elastic recovery capacity of the various samples. The MSCR tests were conducted in accordance with ASTM D7405 [21], with the test temperature set at 64 °C; 10 loading–unloading cycles were applied at two stress levels, 0.1 kPa and 3.2 kPa, to obtain parameters such as Jnr0.1, Jnr3.2, R0.1 and R3.2. It should be noted that although the samples did not undergo a standard ageing procedure, the thermal treatment at 180 °C during preparation may still have had some effect on the state of the binder; therefore, this study focuses more on the relative differences between the various formulations.

### 2.7. BBR Test

To compare the creep response and stress relaxation behaviour of modified bitumen under low-temperature conditions, tests were conducted using a bending beam rheometer (BBR). The tests were carried out in accordance with ASTM D6648 [22], at test temperatures of −12, −18 and −24 °C. The creep stiffness S and the m value were recorded at 60 s; S reflects the material’s ability to resist deformation at low temperatures, whilst the m value characterises the material’s stress relaxation level. These two parameters were combined to evaluate the low-temperature crack resistance potential of different formulations [23].

### 2.8. Fourier Transform Infrared Spectroscopy (FTIR)

Fourier Transform Infrared Spectroscopy (FTIR) was employed to compare changes in characteristic absorption between the base bitumen and various modified samples, with a particular focus on the relative differences in peak positions and intensities following the introduction of polymers and chemical additives. In this study, FTIR was employed as a supplementary structural characterisation technique to analyse internal interactions and trends in structural changes within the system, rather than being used in isolation as a direct criterion for determining whether specific chemical cross-linking had occurred. In subsequent analyses, the FTIR results were primarily discussed in conjunction with rheological properties, storage stability and microstructural morphology [24,25].

### 2.9. Fluorescence Microscopy and Storage Stability Test

In order to assess the compatibility of the system from both a microscopic phase and a macroscopic hierarchical perspective, this study employed fluorescence microscopy to observe the dispersion morphology of the samples and conducted storage stability tests in accordance with ASTM D7173 [26]. Under the fluorescence microscope, regions enriched with polymer appear as bright areas, whilst the continuous bitumen phase appears as relatively darker regions; this allows for a direct comparison of the distribution of the polymer phase across the different sample groups. In the storage stability test, approximately 50 g of modified bitumen was placed in a standard aluminium tube and left to stand at 163 °C for 48 h. It was then cooled, set at a low temperature, and cut into three sections along its length; samples from the upper and lower sections were taken to determine the softening point. The difference in softening points between the upper and lower sections (SPD) is used as an indicator of the degree of phase separation; a smaller SPD indicates that the system is more stable during high-temperature standing and has a weaker tendency for phase separation [26].

### 2.10. Scanning Electron Microscope Test (SEM)

To further compare the surface morphology and dispersion characteristics of the matrix asphalt, control samples and orthogonally modified samples, observations were carried out using a Scanning Electron Microscope (SEM). After cutting, the specimens were mounted on the sample stage; following sputtering treatment, surface scans were performed in secondary electron mode at magnifications of 300× and 1000×. The SEM results were primarily used to assist in identifying microstructural differences between the various formulations and were analysed in conjunction with FTIR and fluorescence microscopy results to assess changes in sample compatibility [27].

## 3. Results and Discussion

### 3.1. Orthogonal Experiments and Conventional Physical Properties

#### 3.1.1. Conventional Physical Properties

To compare the effects of polymer blending and subsequent chemical additives on the basic properties of the material, two sets of control samples—4P+4E and 4P+4E+2S—were prepared during the process; their conventional physical properties are shown in Table 5. As shown in Table 5, the addition of 2% SBS to the 4P+4E system resulted in an increase in both the softening point and the elongation at 5 °C, whilst the penetration value decreased further. This indicates that the introduction of SBS helps to enhance the material’s high-temperature stability and improve its low-temperature deformation resistance to some extent. However, the difference in softening points between the two sets of control samples remains significant, indicating that the blending of PE, EVA and SBS alone is insufficient to effectively suppress the delamination phenomenon during high-temperature standing.

**Figure 2:**
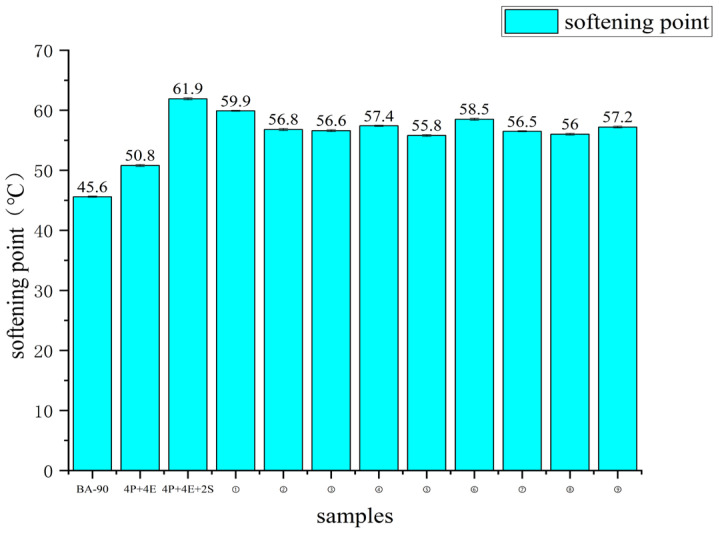

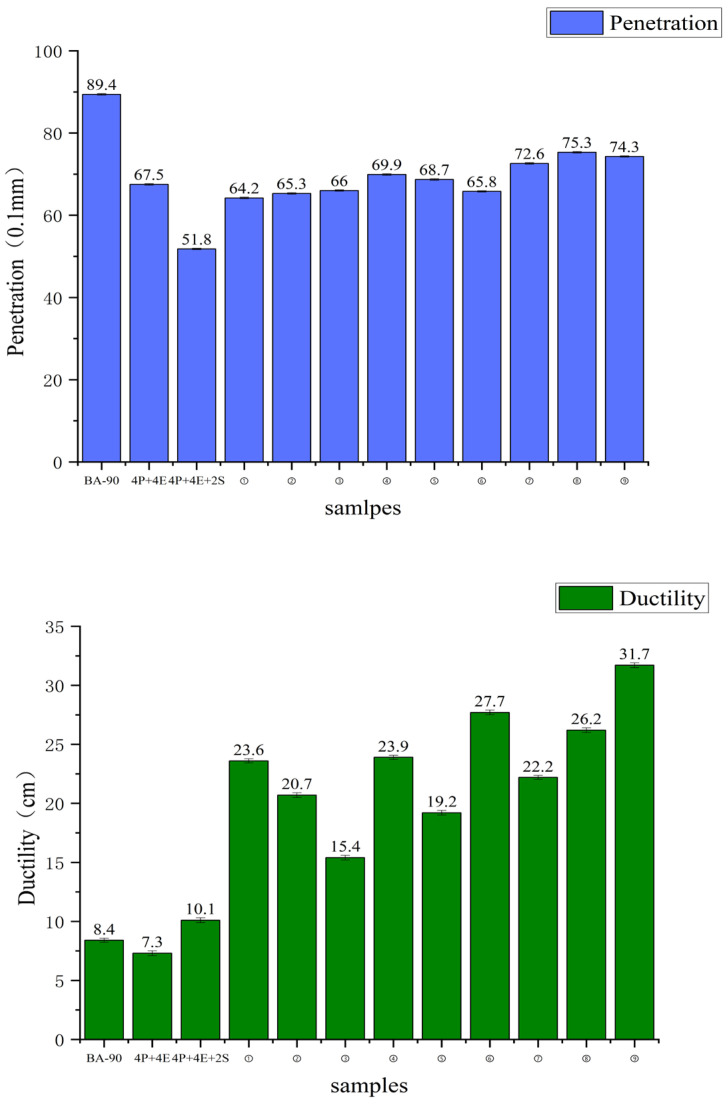
Conventional properties of asphalt binders: softening point, ductility at 5 °C, and penetration at 25 °C. Data are presented as mean ± SD (*n* = 3).

Figure 2 shows the results for the softening point, 5 °C elongation and 25 °C penetration of nine sets of orthogonal samples. Compared with the 4P+4E formulation, the softening point of the orthogonal samples increased by 9.8% to 17.9%, and the 5 °C elongation increased by 110.9% to 334.2%; When compared to the 4P+4E+2S reference, the softening points of the orthogonal groups decreased by 3.2% to 9.9%, yet the 5 °C elongation still increased by 52.5% to 213.9%. This indicates that whilst maintaining a relatively high softening point, the additive combinations produced a more pronounced improvement in low-temperature elongation capacity. The penetration results in Figure 2 also indicate that the penetration of all modified samples was lower than that of the base asphalt; following the introduction of chemical additives, the penetration value increased slightly compared to the 4P+4E+2S control group. Considering the formulation characteristics, this change is more likely related to the regulation of binder fluidity by FEO and the influence of additives on the dispersion state of the polymer phase, rather than being directly attributed to the result of a specific reaction mechanism. For conciseness, Figure 2 is retained mainly for visual comparison, whereas the detailed orthogonal range-analysis results are reported in Table 6, Table 7 and Table 8.

#### 3.1.2. Orthogonal Experiments Results

Table 6, Table 7 and Table 8 present the results of the range analysis for penetration, softening point and 5 °C elongation, respectively. The results show that FEO has the most significant effect on penetration, with a range value markedly higher than that of other factors, indicating that this component has the strongest effect on regulating the consistency of the material. With regard to the softening point, the stabiliser has the greatest influence, followed by elemental sulphur; whereas for the 5 °C elongation, the effects of the stabiliser and FEO are more pronounced. Overall, as the FEO content increases, both penetration and ductility show an upward trend, whilst the softening point decreases; conversely, an increase in stabiliser content results in the opposite trend, namely an increase in the softening point and a decrease in ductility. It can thus be seen that, among the factors examined in this study, FEO tends to improve the material’s flowability and low-temperature deformation capacity, whereas the stabiliser primarily enhances high-temperature stability.

Note: K1–K3 denote the sums at levels 1–3, k1–k3 denote the corresponding averages, and R (RA–RD for factors A–D) represents the range used to evaluate factor influence. This notation is used consistently in Table 6, Table 7, Table 8 and Table 9.

#### 3.1.3. The Penetration Index of Asphalt

**Figure 3:**
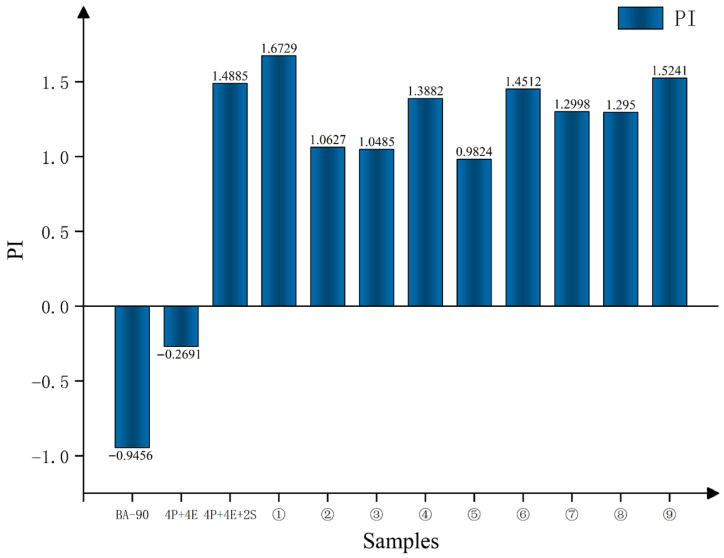
The penetration index (PI) of different samples.

The penetration index (PI), calculated from the penetration at 25 °C and the ring-and-ball softening point using Equation (2), was used to assess the temperature susceptibility of the binders. The PI values of all samples are presented in Figure 3.

(2) $$PI=\frac{1952-500\;\mathrm{lg}P_{25}-20T_{R\&B}}{50\;\mathrm{lg}P_{25}-T_{R\&B}-120}$$

where

lgP25 is the needle penetration (25 °C);

TR&B is the softening point.

Compared with BA-90, the PI of the control samples increased significantly following the addition of PE and EVA; on this basis, the addition of 2% SBS further increased the PI, indicating that the sensitivity of the material to temperature changes was reduced following the formulation of the ternary polymer blend. The PI of all nine orthogonal samples was higher than that of BA-90 and 4P+4E; samples No. 1 and No. 9 reached 1.6729 and 1.5241, respectively, which were also higher than the 4P+4E+2S control group. Combining Figure 2 and Table 5, it can be seen that the introduction of chemical additives not only altered the relationship between the material’s penetration and softening point but also further improved its thermal stability.

### 3.2. Rotational Viscosity

**Figure 4:**
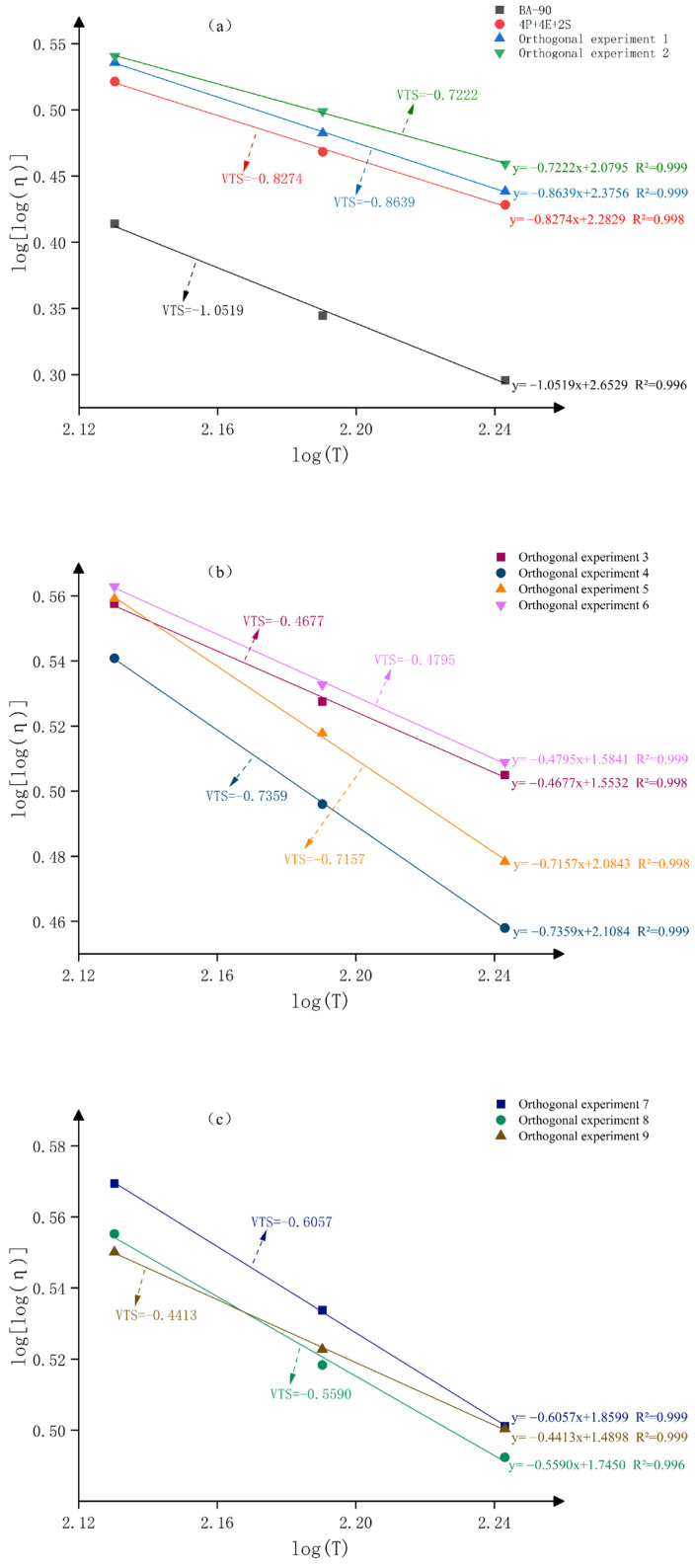
The Viscosity–temperature curves for each asphalt sample. (**a**) BA-90, orthogonal experiment1, 4P+4E+2S, orthogonal experiment2; (**b**) orthogonal experiment3, orthogonal experiment4, orthogonal experiment5, orthogonal experiment6; (**c**) orthogonal experiment7, orthogonal experiment8, orthogonal experiment9.

Figure 4 shows the viscosity–temperature relationships for the different samples. BA-90 exhibits the lowest VTS value of −1.0519, indicating that its viscosity varies significantly with temperature. Following the addition of PE, EVA and SBS, the VTS value of the 4P+4E+2S control group increased markedly, rising by 74.8% compared to BA-90, suggesting that polymer blending can reduce the binder’s sensitivity to temperature changes. On this basis, the VTS values of the nine orthogonal samples were further improved, all exceeding those of BA-90 and 4P+4E+2S; sample No. 8 reached −0.4413, representing increases of 58.0% and 46.7% compared to BA-90 and 4P+4E+2S, respectively. This result indicates that, following the introduction of FEO, elemental sulphur, stabilisers and Z-6036 into the ternary polymer system, the material’s high-temperature viscosity response stabilised further.

### 3.3. The Dynamic Shear Rheometer Test

#### 3.3.1. Temperature Sweep

**Figure 5:**
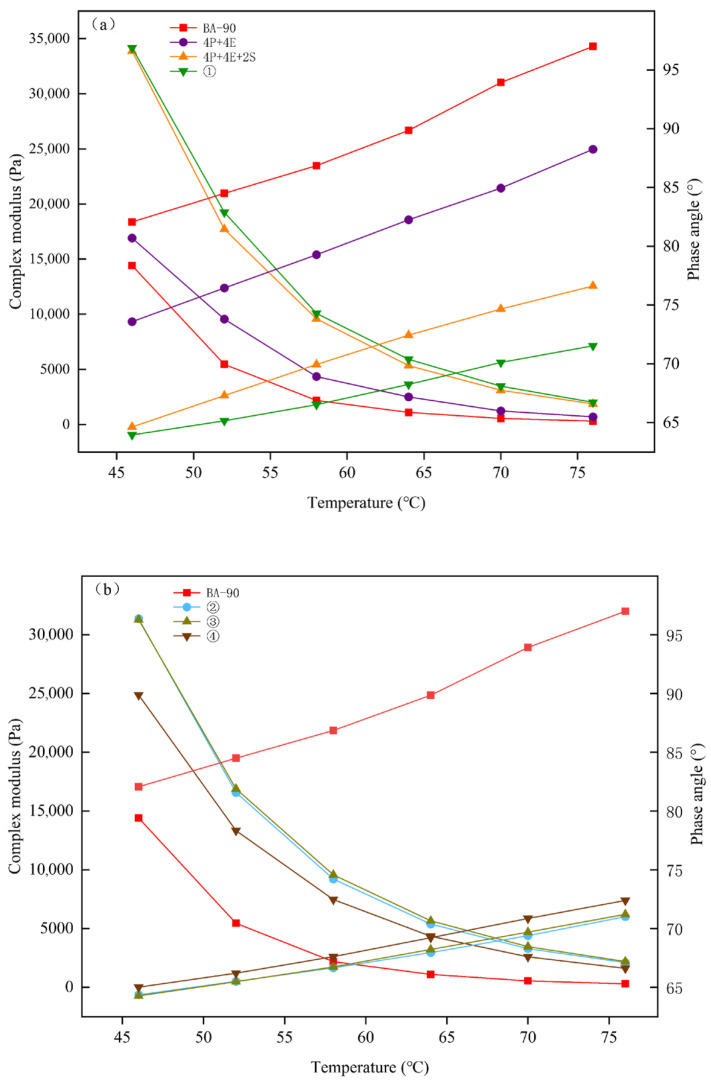

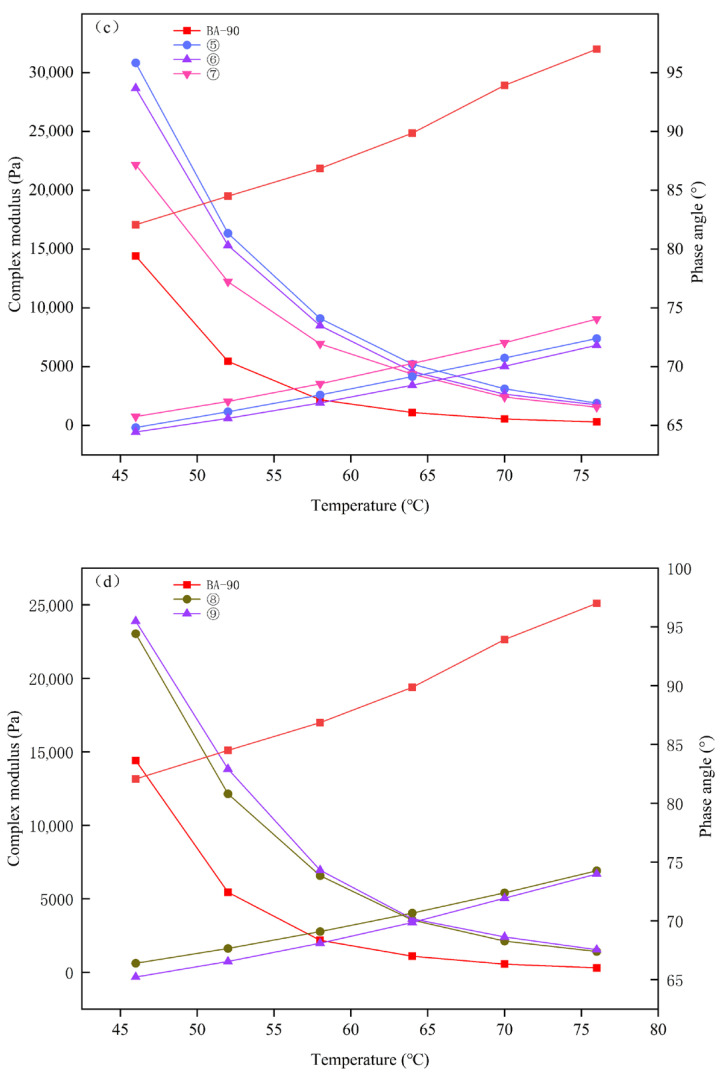
|G*| and δ for each asphalt binder sample. (**a**) BA-90,4P+4E,4P+4E+2S, orthogonal experiment1; (**b**) orthogonal experiment2, orthogonal experiment3, orthogonal experiment4; (**c**) orthogonal experiment5, orthogonal experiment6, orthogonal experiment7; (**d**) orthogonal experiment8,orthogonal experiment9.

**Figure 6:**
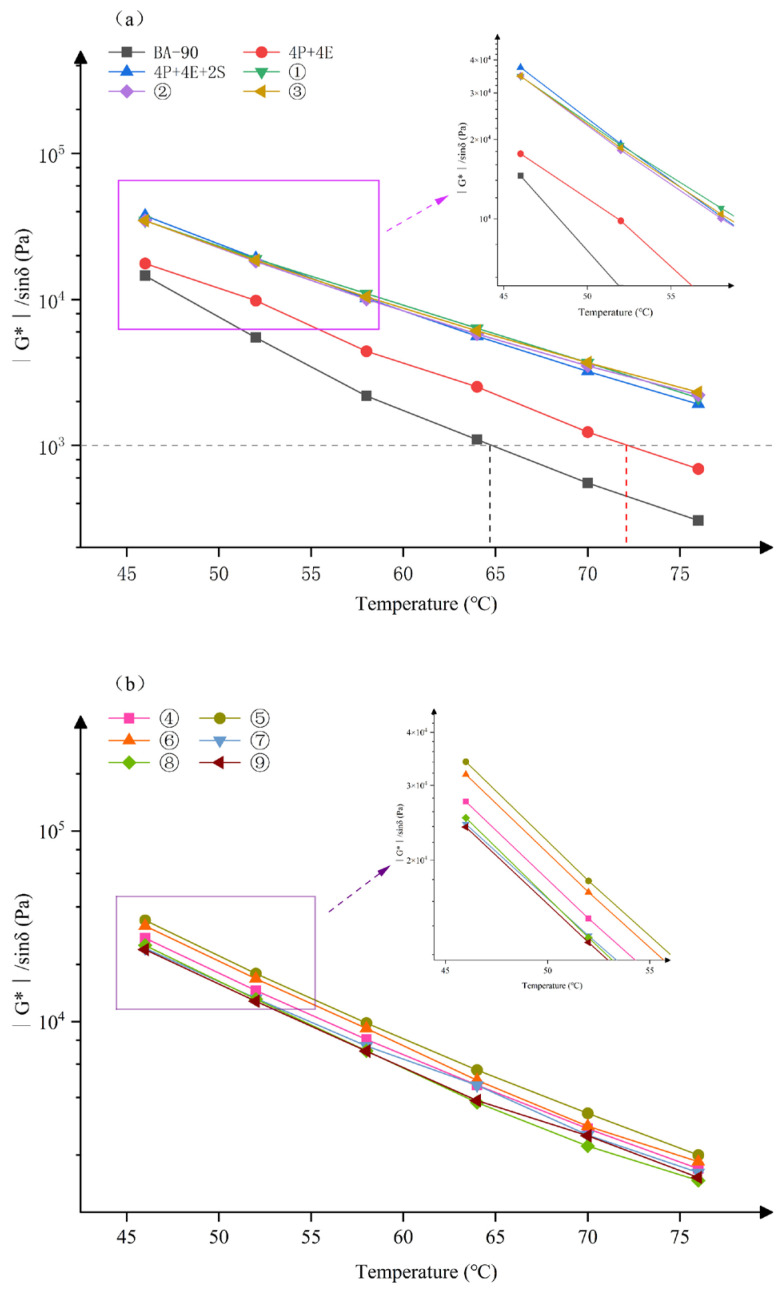
Rutting factors of different modified asphalt samples. (**a**) BA-90, 4P+4E, 4P+4E+2S, orthogonal experiment1, orthogonal experiment2, orthogonal experiment3; (**b**) orthogonal experiment4, orthogonal experiment5, orthogonal experiment6, orthogonal experiment7, orthogonal experiment8, orthogonal experiment9.

Figure 5 and Figure 6 illustrate the differences in viscoelastic response between various samples under temperature-sweep conditions. Compared with BA-90, both 4P+4E and 4P+4E+2S exhibited higher complex modulus G* and lower phase angle δ at the same temperature, indicating that the polymer blend maintains greater structural rigidity under high-temperature shear stress and exhibits enhanced elastic properties. Following the addition of chemical additives to this formulation, the G* of most orthogonal samples increased further, whilst δ decreased further, indicating that the system’s resistance to shear deformation under high-temperature conditions was enhanced following formulation adjustment.

The changes in the rutting factor shown in Figure 6 are consistent with the above results. The incorporation of PE and EVA significantly increased the G*/sinδ of the samples, and this value continued to rise following the addition of SBS. Taking 52 °C as an example, the rutting factors of Samples 1, 2 and 3 were higher than those of the 4P+4E+2S control group. Meanwhile, the failure temperatures of BA-90 and 4P+4E were 64.7 °C and 72.1 °C, respectively, whereas the 4P+4E+2S and the nine orthogonal samples all maintained a G*/sinδ value greater than 1.0 kPa within the test range. Overall, the ternary polymer blend has already significantly improved the material’s high-temperature rutting resistance, whilst the further addition of chemical additives has sustained and enhanced this advantage. It should be noted that as the temperature rises, all samples exhibit a decrease in G* and an increase in δ; this is a regular change commonly observed in bituminous binders at high temperatures. Therefore, the focus of this section should be on the relative differences between the various formulations, rather than this general characteristic itself.

#### 3.3.2. Multiple Stress Creep and Recovery

**Figure 7:**
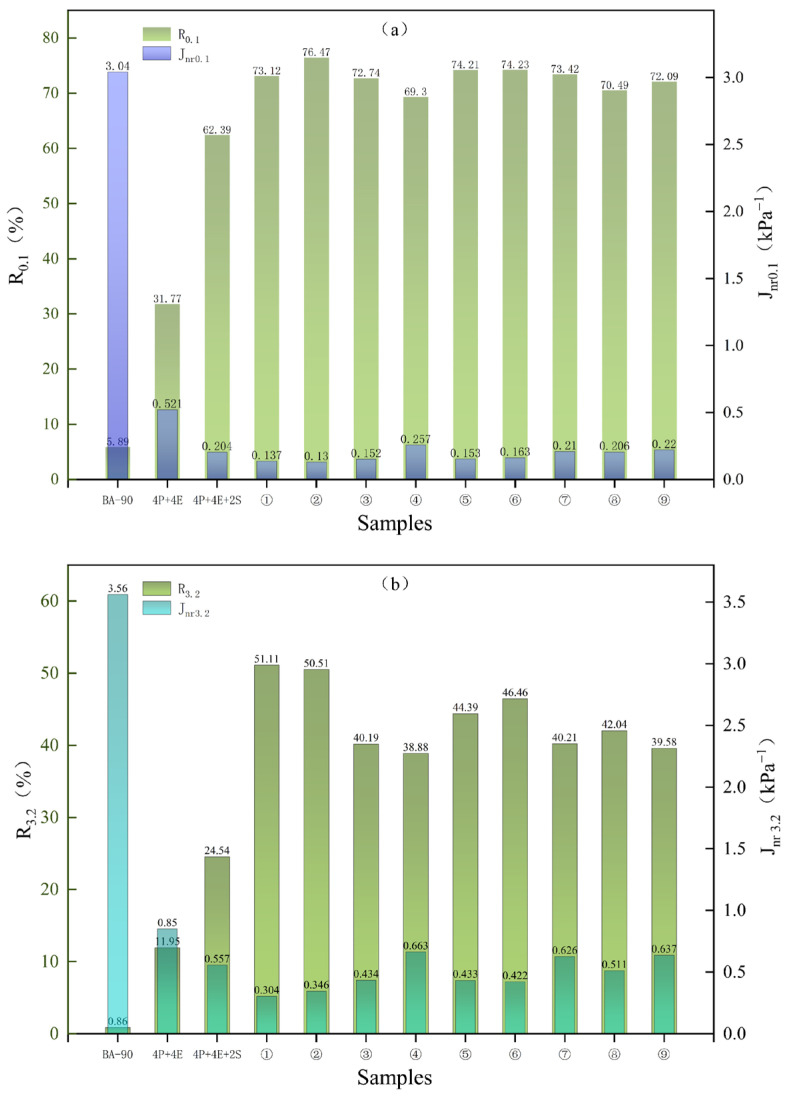
Irrecoverable creep flexibility of modified asphalt under different shear stresses: (**a**) under the stress of 0.1 kPa; (**b**) under the stress of 3.2 kPa.

Figure 7 shows the multi-stress creep recovery results for different samples at 64 °C. As can be seen from the figure, at both stress levels of 0.1 kPa and 3.2 kPa, the irreversible creep modulus of the 4P+4E and 4P+4E+2S samples was lower than that of BA-90, indicating that the ability of the material to resist permanent deformation has been improved following polymer blending; simultaneously, the irreversible creep modulus of all samples at 3.2 kPa is higher than that at 0.1 kPa, indicating that the material is more prone to irreversible deformation as the stress increases.

For the nine orthogonal sample groups, the irreversible creep modulus at both stress levels was generally lower than that of BA-90 and 4P+4E; the improvement observed in the control group following the addition of SBS was also replicated in the orthogonal groups. In particular, samples 5 and 6 showed better results than 4P+4E+2S at both 0.1 kPa and 3.2 kPa, indicating that the corresponding formulations are less prone to cumulative residual deformation under repeated loading. The recovery rates showed an inverse trend. Following the addition of 4% PE and 4% EVA, the recovery rates of the samples at 0.1 kPa and 3.2 kPa increased from 5.89% and 0.86% to 31.77% and 11.95%, respectively; further addition of 2% SBS resulted in a further increase in recovery rates to 62.39% and 24.54%. The recovery rates of all nine orthogonal samples were higher than those of the matrix asphalt and the two control samples, indicating that the introduction of chemical additives not only reduces irreversible deformation but also enhances the material’s ability to recover after load removal.

### 3.4. Low-Temperature Properties Analysis

**Figure 8:**
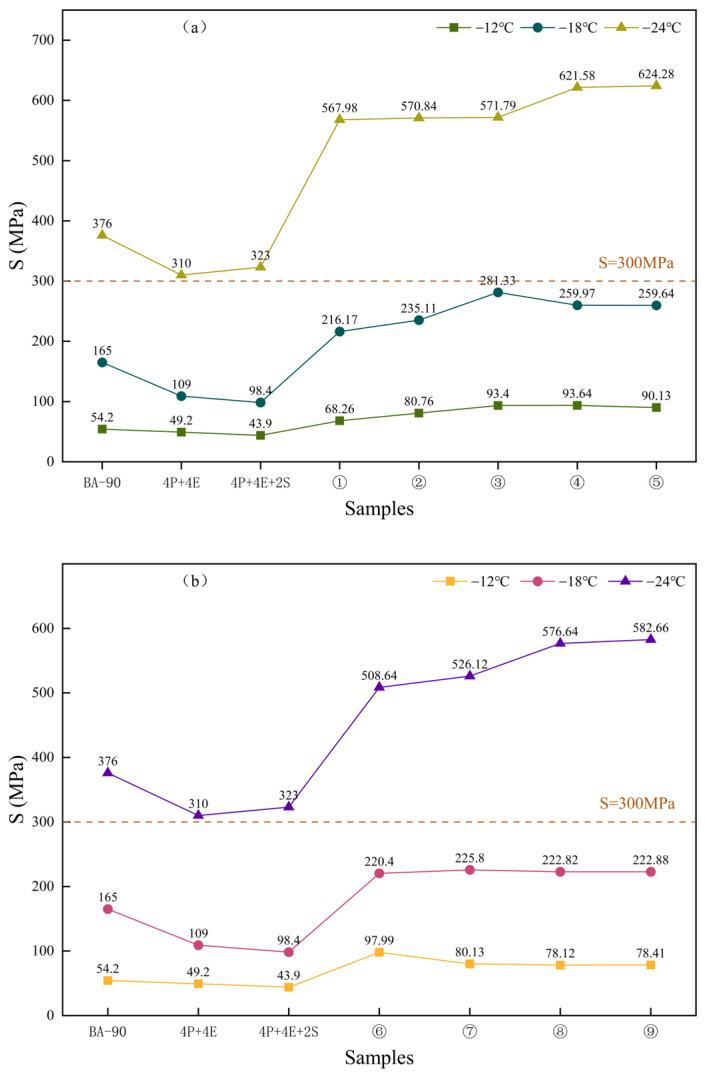
The creep stiffness modulus of asphalt binder samples at various temperatures. (**a**) BA-90, 4P+4E, 4P+4E+2S, orthogonal experiment1, orthogonal experiment2, orthogonal experiment3, orthogonal experiment4, orthogonal experiment5. (**b**) orthogonal experiment6, orthogonal experiment7, orthogonal experiment8, orthogonal experiment9.

**Figure 9:**
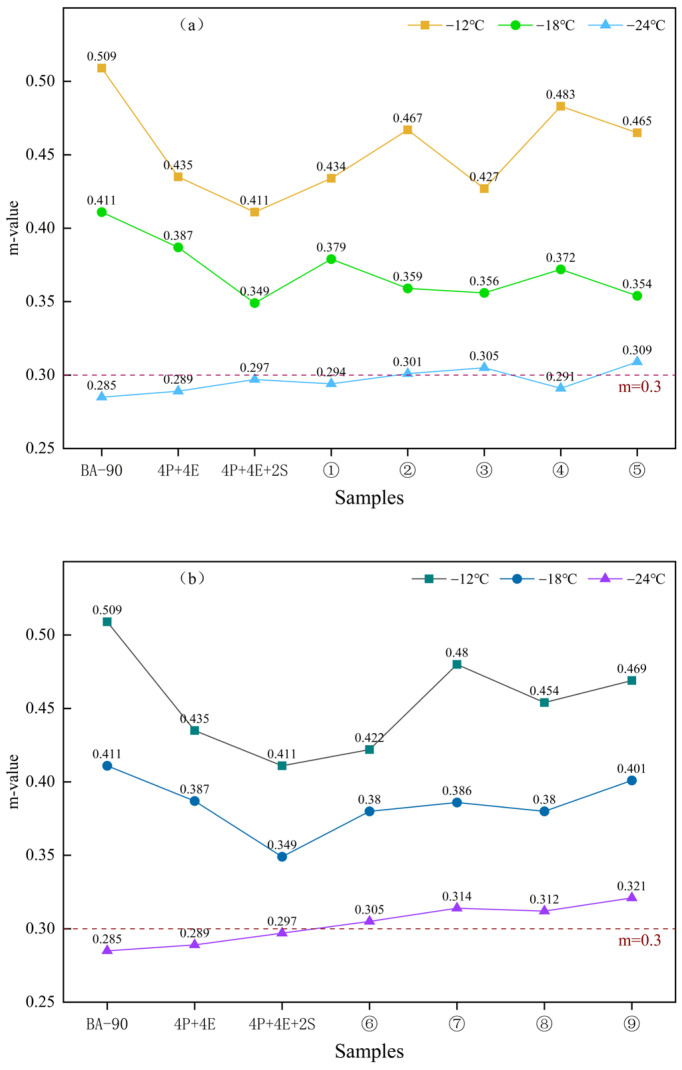
The rate of change in creep strength of asphalt binder samples at each temperature. (**a**) BA-90, 4P+4E, 4P+4E+2S, orthogonal experiment1, orthogonal experiment2, orthogonal experiment3, orthogonal experiment4, orthogonal experiment5. (**b**) orthogonal experiment6, orthogonal experiment7, orthogonal experiment8, orthogonal experiment9.

Figure 8 and Figure 9 show the creep stiffness and m-values of the various samples under low-temperature conditions, respectively. As can be seen from Figure 8, the creep stiffness of both sets of control samples at −12 °C, −18 °C and −24 °C was lower than that of BA-90, indicating that the deformation capacity of the material under low-temperature loading has been improved following polymer blending. For the nine samples in the orthogonal group, the creep stiffness was less than 300 MPa at both −12 °C and −18 °C; although none of the samples met the specification requirements at −24 °C, the orthogonal group still performed better overall than the two control groups, indicating that chemical additives have a positive effect on low-temperature crack resistance potential.

The m-value results shown in Figure 9 further support the above conclusion. At −12 °C, the m-values of all nine orthogonal samples were higher than those of the two control samples, with Sample 9 exhibiting strong stress relaxation behaviour under all three temperature conditions. Overall, seven of the nine orthogonal samples met the requirement of m ≥ 0.3. For samples that did not meet the specification requirements, a more reasonable explanation would be insufficient formulation balance or limited compatibility, rather than directly attributing this to the formation of a specific cross-linked structure. Combining Figure 8 and Figure 9, it can be concluded that the addition of additives improved the synergy between the polymer phase and the bitumen phase, enabling the system to exhibit better deformation and relaxation capabilities at low temperatures; however, significant differences still exist between different formulations.

### 3.5. Fourier Transform Infrared Spectroscopy Test (FTIR)

**Figure 10:**
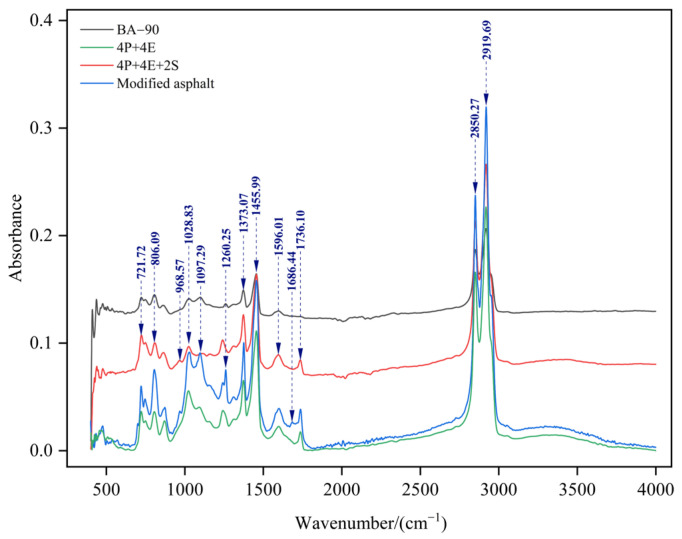
The FTIR spectral analysis curves of different asphalt binders.

Figure 10 presents the infrared spectra of the base binder, the control binders, and the orthogonally designed binders. Compared with BA-90, the modified binders showed noticeable differences in several characteristic absorption regions [28,29]. After SBS was introduced, identifiable absorptions appeared near 968.57 cm^−1^ and 700.51 cm^−1^; in the PE/EVA-containing system, changes around 1260.25 cm^−1^ and 1736.10 cm^−1^ became more evident; after the chemical additives were incorporated, further variations could also be observed near 1028.83 cm^−1^ as well as around 1455.99, 2850.27 and 2919.69 cm^−1^. These spectral differences indicate that the incorporation of polymers and additives altered the compositional features of the system and modified its local structural environment.

In this section, the emphasis is placed on the relative differences among formulations, including whether characteristic peaks appear, whether their intensities change, and whether those changes remain consistent across different samples, rather than on an exhaustive interpretation of every absorption band. Taken together, the spectra suggest that the combined incorporation of PE, EVA, SBS, and the subsequent additives changed the internal interactions among the components and made the response of several characteristic absorption regions more apparent. Accordingly, FTIR is used here as auxiliary evidence for structural variation and dispersion adjustment, whereas the spectra alone are insufficient to confirm the formation of any specific chemical cross-linking structure.

### 3.6. Fluorescence Microscopy and Storage Stability Test

**Figure 11:**
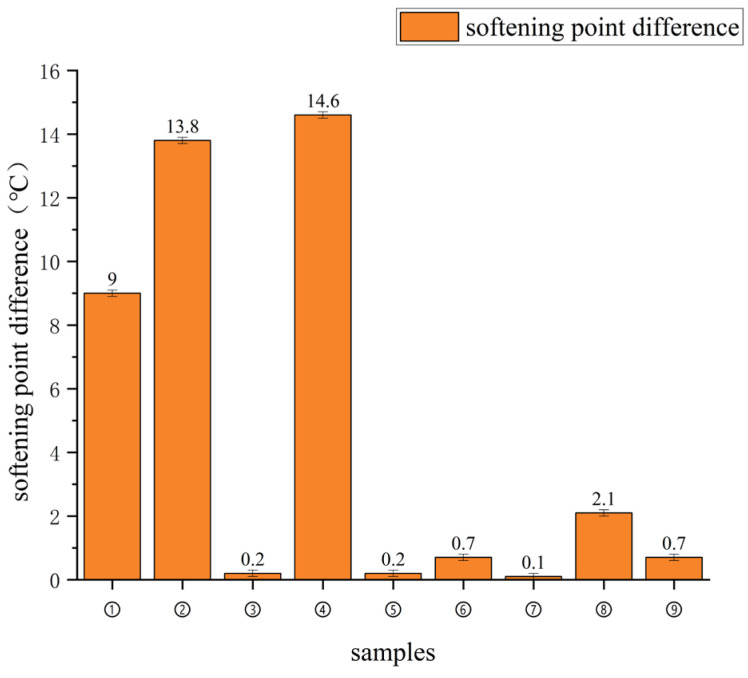
Softening point difference (segregation) results of orthogonal asphalt binder samples. Data are presented as mean ± SD (*n* = 3).

Table 9 and Figure 11 summarise the softening point differences in the orthogonal samples. Table 9 provides the detailed range-analysis results, whereas Figure 11 is retained only as a visual summary of the segregation behaviour. For the nine orthogonally designed binders, the softening point difference ranged from 14.6 °C to 0.1 °C, whereas the corresponding values of the two control binders, 4P+4E and 4P+4E+2S, were 33.4 °C and 35.2 °C, respectively. These results show that, after FEO, elemental sulphur, stabiliser and Z-6036 were introduced into the ternary polymer system, the degree of segregation under high-temperature static conditions was reduced overall. Among the nine orthogonal samples, six satisfied the requirement of SPD ≤ 2.5 °C, indicating that acceptable storage stability was achieved in a portion of the formulations.

**Figure 12:**
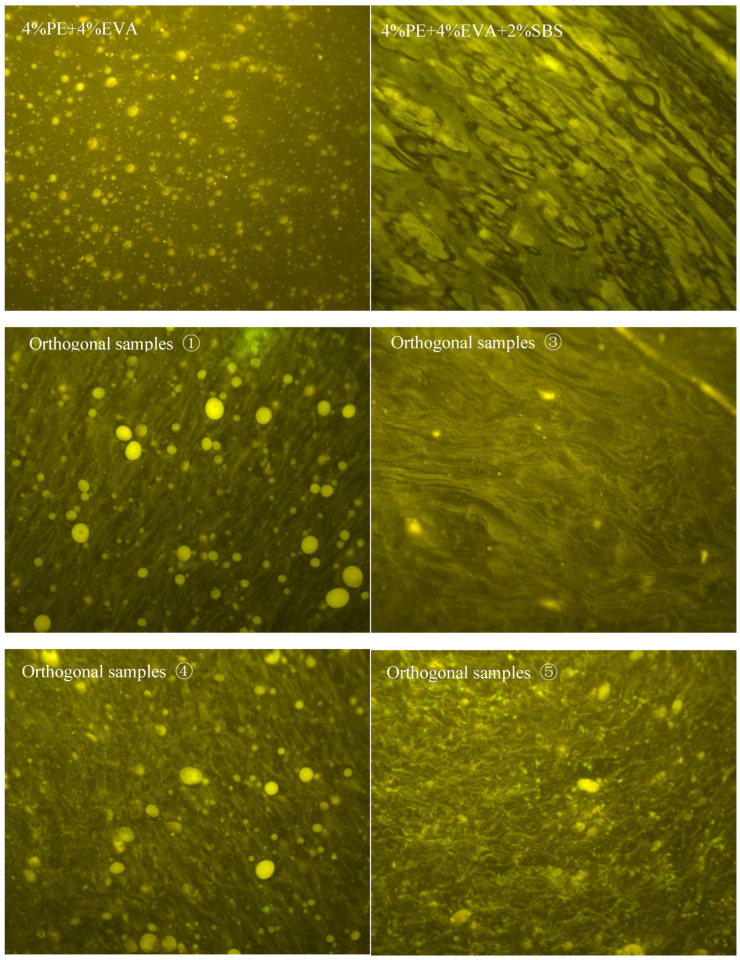
Fluorescence microscopy images of each asphalt binder.

Figure 12 further shows the distribution characteristics of the polymer-rich phase in different binders. In the 4% PE + 4% EVA sample, the polymer-rich phase mainly appeared as relatively scattered particles within the continuous bitumen phase, and local aggregation was still evident. After 2% SBS was added, the polymer-rich zones began to show a certain degree of connectivity, indicating that SBS changed the original phase-distribution pattern. For orthogonally modified samples ①, ③, ④ and ⑤, the continuity of the polymer-rich regions was further improved, although the extent of improvement differed among formulations. Samples ① and ④ still showed obvious local agglomeration, whereas in samples ③ and ⑤, the number of isolated particles was markedly reduced and the overall distribution became more uniform. Similar morphology-based observations have also been reported for other polymer-modified binder systems [14,30].

When the softening point differences are interpreted together with the fluorescence images, samples ①, ③, ④ and ⑤ exhibited values of 9.0 °C, 0.2 °C, 14.6 °C and 0.2 °C, respectively, all lower than the 35.2 °C measured for 4P+4E+2S. Among them, samples ③ and ⑤ met the storage-stability criterion, whereas samples ① and ④, although clearly improved relative to the control binder, still did not satisfy the specification. This comparison indicates that the introduction of chemical additives can reduce the tendency toward phase separation, but the magnitude of the improvement remains formulation-dependent. Taken together, the softening point difference and fluorescence results support a reduction in phase separation and a more favourable phase distribution, but they should not be interpreted as direct proof that a specific cross-linking reaction has occurred.

### 3.7. Scanning Electron Microscope Test (SEM)

**Figure 13:**
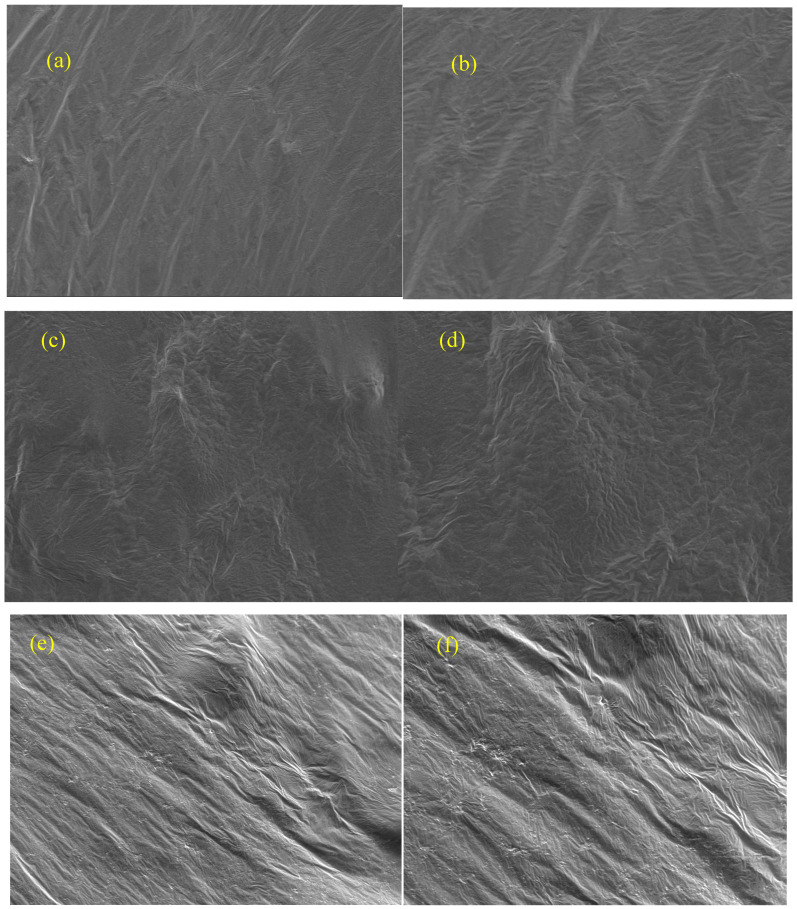
The Scanning Electron Microscope images of different asphalt samples. (**a**) BA-90; (**b**) 4P+4E+2S; (**c**) orthogonal experiment1; (**d**) orthogonal experiment3; (**e**) orthogonal experiment4; (**f**) orthogonal experiment5.

Figure 13 presents SEM images of different binders at magnifications of 300× and 1000×. The surface of BA-90 was comparatively even, without obvious interconnected phase features. After polymer blending, the control binder displayed polymer-phase features with different characteristic sizes, suggesting that a certain degree of dispersion non-uniformity still remained in the system. By contrast, the orthogonally modified binder showed fewer isolated particle features and, in some local regions, a more continuous surface morphology.

This morphological change is generally consistent with the phase-distribution trend observed in Figure 12 and suggests that the chemical additives positively influenced the distribution state of the polymer-rich phase. In other words, after the additives were introduced, the continuity of the polymer-rich domains increased, and the internal structure of the binder became comparatively more stable. SEM mainly provides morphology-level information [14,27]; its role here is to assist in judging whether the system became more homogeneous and whether obvious phase separation was alleviated. Therefore, the SEM observations are better used to support improved microstructural uniformity and compatibility than to infer a specific bonding mode or a definite chemical cross-linking structure.

## 4. Conclusions

Focusing on a ternary composite system comprising SBS/PE/EVA-modified bitumen and chemical additives, this paper compares different formulations using orthogonal design. By combining macroscopic performance testing with microscopic characterisation, the following conclusions have been reached.

1. Following the incorporation of FEO, elemental sulphur, stabilisers and Z-6036 into the PE/EVA/SBS ternary composite system, the routine properties of the samples were significantly optimised. Compared with the 4P+4E+2S control group, the orthogonal group maintained a high softening point whilst showing a marked improvement in elongation at 5 °C and a slight increase in penetration. This indicates that the addition of chemical additives helps to improve the material’s low-temperature elongation capacity and regulate its basic viscosity level.

2. The results of rotational viscosity, DSR and MSCR tests indicate that the high-temperature rheological properties of the material were further improved following the addition of additives. The VTS values of the orthogonal group samples were generally higher than those of the matrix bitumen and the control group, suggesting a reduced viscosity–temperature sensitivity; at the same time, some samples exhibited higher rutting factors, lower irreversible creep modulus and higher recovery rates, indicating that they are less prone to permanent deformation under high-temperature loading.

3. The BBR results indicate that chemical additives have a positive effect on the low-temperature behaviour of ternary composite modified bitumen. Compared with the control group, the samples in the orthogonal group exhibited lower overall creep stiffness and higher m-values at −12 °C and −18 °C; the majority of these samples met the requirement of m ≥ 0.3, suggesting that their low-temperature stress relaxation capacity and crack resistance potential have been enhanced.

4. The results from softening point difference measurements, fluorescence microscopy, SEM and FTIR analysis collectively indicate that the addition of the additive reduces the system’s tendency towards phase separation, leads to a more uniform distribution of the polymer phase, and improves microstructural stability. Notably, the softening point difference in some orthogonal samples fell below 2.5 °C, demonstrating good storage stability. The microstructural characterisation results support the conclusion that ‘compatibility has improved, the dispersion state has been optimised and internal interactions within the system have been enhanced’; however, the available evidence is still insufficient to prove, on its own, that a specific form of chemical cross-linking reaction has occurred.

## Figures and Tables

**Table 1:** General physical properties of BA-90.

Items	Test Temperature (°C)	Value
Penetration (0.1 mm)	25	89.4
Softening point (°C)	-	45.6
Ductility (mm)	5	84.0
Viscosity (Pa/s)	135	0.370

**Table 2:** Properties of PE and EVA copolymers.

Property	Value	
	PE	EVA
Density at 25 °C (g/cm^3^)	0.925	0.948
Melting point (°C)	100	75
Melt Index (g/10 min)	70	52

**Table 3:** Factors and levels in orthogonal tests.

Levels	Factors			
	A	B	C	D
	FEO	S	Stabiliser	Z-6036
	(g)	(g)	(g)	(g)
1	10	0.40	0.75	0.10
2	15	0.50	1.00	0.30
3	20	0.60	1.25	0.50

**Table 4:** Orthogonal experiment plan.

	Factors				
Test Number	A	B	C	D	Test Program
①	1	1	1	1	A1B1C1D1
②	1	2	2	2	A1B2C2D2
③	1	3	3	3	A1B3C3D3
④	2	1	2	3	A2B1C2D3
⑤	2	2	3	1	A2B2C3D1
⑥	2	3	1	2	A2B3C1D2
⑦	3	1	3	2	A3B1C3D2
⑧	3	2	1	3	A3B2C1D3
⑨	3	3	2	1	A3B3C2D1

**Table 5:** Conventional physical properties of 4P+4E and 4P+4E+2S modified asphalts.

Items	Test Temperature (°C)	Value	
		4P+4E	4P+4E+2S
Penetration (0.1 mm)	25	67.5	51.8
Softening point (°C)	-	50.8	61.9
Ductility (cm)	5	7.3	10.1
SPD (°C)	-	33.4	35.2

**Table 6:** Orthogonal range-analysis results for softening point.

	Factors			
Test Number	A	B	C	D
①	1	1	1	1
②	1	2	2	2
③	1	3	3	3
④	2	1	2	3
⑤	2	2	3	1
⑥	2	3	1	2
⑦	3	1	3	2
⑧	3	2	1	3
⑨	3	3	2	1
K1	173.3	173.8	174.4	172.9
K2	171.7	168.6	171.4	171.8
K3	169.7	172.3	168.9	170.0
k1	57.8	57.9	58.1	57.6
k2	57.2	56.2	57.1	57.3
k3	56.6	57.4	56.3	56.7
R	1.2	1.7	1.8	0.9
RC > RB > RA > RD				

**Table 7:** Orthogonal range-analysis results for ductility at 5 °C.

	Factors			
Test Number	A	B	C	D
①	1	1	1	1
②	1	2	2	2
③	1	3	3	3
④	2	1	2	3
⑤	2	2	3	1
⑥	2	3	1	2
⑦	3	1	3	2
⑧	3	2	1	3
⑨	3	3	2	1
K1	59.7	69.7	77.5	74.5
K2	70.8	66.1	76.3	70.6
K3	80.1	74.8	56.8	65.5
k1	19.9	23.2	25.8	24.8
k2	23.6	22.0	25.4	23.5
k3	26.7	24.9	18.9	21.8
R	6.8	2.9	6.9	3.0
RC > RA > RD > RB				

**Table 8:** Orthogonal range-analysis results for penetration at 25 °C.

	Factors			
Test Number	A	B	C	D
①	1	1	1	1
②	1	2	2	2
③	1	3	3	3
④	2	1	2	3
⑤	2	2	3	1
⑥	2	3	1	2
⑦	3	1	3	2
⑧	3	2	1	3
⑨	3	3	2	1
K1	195.5	206.7	205.3	207.2
K2	204.4	209.3	209.5	203.7
K3	222.2	206.1	207.3	211.2
k1	65.2	68.9	68.4	69.1
k2	68.1	69.8	69.8	67.9
k3	74.1	68.7	69.1	70.4
R	8.9	1.1	1.4	2.5
RA > RD > RC > RB				

**Table 9:** Orthogonal range-analysis results for softening point difference.

	Factors			
Test Number	A	B	C	D
①	1	1	1	1
②	1	2	2	2
③	1	3	3	3
④	2	1	2	3
⑤	2	2	3	1
⑥	2	3	1	2
⑦	3	1	3	2
⑧	3	2	1	3
⑨	3	3	2	1
K1	23.0	23.7	11.8	9.9
K2	15.5	16.1	29.1	14.6
K3	2.9	1.6	0.5	16.9
k1	7.7	7.9	3.9	3.3
k2	5.2	5.4	9.7	4.9
k3	1.0	0.5	0.2	5.6
R	6.7	7.4	9.5	2.3
RC > RB > RA > RD				

## Data Availability

The original contributions presented in this study are included in the article. Further inquiries can be directed to the corresponding author.

## Abbreviations

The following abbreviations are used in this manuscript:

PE	Polyethylene
LLDPE	Linear low-density polyethylene
LDPE	Low-density polyethylene
EVA	Ethylene vinyl acetate
SBS	Styrene butadiene styrene
FEO	Furfural Extract Oil
Z-6036	3-(Triethoxysilyl)propyl methacrylate
FM	Fluorescence Microscopy
SEM	Scanning Electron Microscope
FTIR	Fourier Transform Infrared Spectrometer
SPD	Softening Point Difference
PI	Penetration Index
4P+4E	4%PE+4%EVA Modified Asphalt
4P+4E+2S	4%PE+4%EVA+2%SBS Modified Asphalt

## References

[1] Munera J., Ossa E. (2014). Polymer modified bitumen: Optimization and selection. Mater. Des. (1980–2015).

[2] Jin X., Guo N., You Z., Wang L., Wen Y., Tan Y. (2020). Rheological properties and micro-characteristics of polyurethane composite modified asphalt. Constr. Build. Mater..

[3] Li Z., Guo T., Chen Y., Liu Q., Chen Y. (2021). The properties of nano-CaCO_3_/nano-ZnO/SBR composite-modified asphalt. Nanotechnol. Rev..

[4] Wen Y., Ma F., Fu Z., Liu J., Dai J., Li C., Dong W., Li S. (2023). Evaluating the low-temperature creep properties of polyphosphoric acid-polymer composite-modified asphalt. Int. J. Pavement Eng..

[5] Li H., Hao G., Zhou L., Wang S., Zhao G., Zhang Y., Temitope A.A. (2023). Effect of different waste plastic modifiers on conventional asphalt performance: Optimal preparation parameters determination and mechanism analysis. Environ. Sci. Pollut. Res..

[6] Liu B., Li J., Han M., Zhang Z., Jiang X. (2020). Properties of polystyrene grafted activated waste rubber powder (PS-ARP) composite SBS modified asphalt. Constr. Build. Mater..

[7] Liu H., Zhang Z., Yu X., Kan S., Luo Y., Han K., Liang Y., Gao J. (2024). Preparation of polyol from waste polyethylene terephthalate (PET) and its application to polyurethane (PU) modified asphalt. Constr. Build. Mater..

[8] Wei K., Wang X., Ma B., Shi W., Duan S., Liu F. (2019). Study on rheological properties and phase-change temperature control of asphalt modified by polyurethane solid–solid phase change material. Sol. Energy.

[9] Yan K., Chen J., You L., Tian S. (2020). Characteristics of compound asphalt modified by waste tire rubber (WTR) and ethylene vinyl acetate (EVA): Conventional, rheological, and microstructural properties. J. Clean. Prod..

[10] Yan K., Xu H., You L. (2015). Rheological properties of asphalts modified by waste tire rubber and reclaimed low density polyethylene. Constr. Build. Mater..

[11] Ma Y., Wang S., Zhou H., Hu W., Polaczyk P., Huang B. (2022). Recycled polyethylene and crumb rubber composites modified asphalt with improved aging resistance and thermal stability. J. Clean. Prod..

[12] Nisar J., Mir M.S. (2023). Study on optimal preparation and rheological characteristics of waste low density polyethylene (LDPE)/styrene butadiene styrene (SBS) composite modified asphalt binder. Constr. Build. Mater..

[13] Xu S., Hu C., Yu J., Que Y., Zhou X. (2017). Performance of mixed asphalt blended with furfural extract oil and its distinction from pure asphalt. Pet. Sci. Technol..

[14] Zhang X., Han C., Otto F., Zhang F. (2021). Evaluation of properties and mechanisms of waste plastic/rubber-modified asphalt. Coatings.

[15] Liu Y., Cao H., Wang Z. (2015). A Kind of SBS Modified Asphalt Stabilizer.

[16] (2014). Standard Test Method for Softening Point of Bitumen (Ring-and-Ball Apparatus).

[17] (2017). Standard Test Method for Ductility of Asphalt Materials.

[18] (2020). Standard Test Method for Penetration of Bituminous Materials.

[19] (2015). Standard Test Method for Viscosity Determination of Asphalt at Elevated Temperatures Using a Rotational Viscometer.

[20] (2015). Standard Test Method for Determining the Rheological Properties of Asphalt Binder Using a Dynamic Shear Rheometer.

[21] (2020). Standard Test Method for Multiple Stress Creep and Recovery (MSCR) of Asphalt Binder Using a Dynamic Shear Rheometer.

[22] (2008). Standard Test Method for Determining the Flexural Creep Stiffness of Asphalt Binder Using the Bending Beam Rheometer (BBR).

[23] Lu Y., Li S., Jiang Y., Yang X., Li L. (2024). Rheological and aging properties of nano-clay/SBS composite-modified asphalt. Materials.

[24] Yang X., Zhu H., Yang S., Tan Q., Wang Y., Huang C. (2024). Surface modification of nano-CaCO_3_ and its enhancement on the performance of SBS/crumb rubber modified asphalt. Constr. Build. Mater..

[25] Zeng G., Shen A., Lyu Z., Kang C., Cui H., Ren G., Yue G. (2023). Research on anti-aging properties of POE/SBS compound-modified asphalt in high-altitude regions. Constr. Build. Mater..

[26] (2020). Standard Practice for Determining the Separation Tendency of Polymer from Polymer-Modified Asphalt.

[27] Huang T., He H., Zhang P., Lv S., Jiang H., Liu H., Peng X. (2022). Laboratory investigation on performance and mechanism of polyphosphoric acid modified bio-asphalt. J. Clean. Prod..

[28] Nciri N., Kim N., Cho N. (2017). New insights into the effects of styrene-butadiene-styrene polymer modifier on the structure, properties, and performance of asphalt binder: The case of AP-5 asphalt and solvent deasphalting pitch. Mater. Chem. Phys..

[29] Wang K., Yuan Y., Han S., Yang Y. (2019). Application of FTIR spectroscopy with solvent-cast film and PLS regression for the quantification of SBS content in modified asphalt. Int. J. Pavement Eng..

[30] Zhang B., Chen H., Zhang H., Kuang D., Wu J., Zhang X. (2019). A study on physical and rheological properties of rubberized bitumen modified by different methods. Materials.

